# Uniportal video-assisted thoracic surgery for major lung resection is associated with less immunochemokine disturbances than multiportal approach

**DOI:** 10.1038/s41598-021-89598-2

**Published:** 2021-05-14

**Authors:** Peter S. Y. Yu, Kin Wai Chan, Rainbow W. H. Lau, Innes Y. P. Wan, George G. Chen, Calvin S. H. Ng

**Affiliations:** 1 Division of Cardiothoracic Surgery, Department of Surgery, Prince of Wales Hospital, The Chinese University of Hong Kong, Shatin, Hong Kong SAR, China; 2 Department of Statistics, The Chinese University of Hong Kong, Shatin, Hong Kong SAR, China; 3 Surgical Research Laboratory, Department of Surgery, Prince of Wales Hospital, The Chinese University of Hong Kong, Shatin, Hong Kong SAR, China; 4 Department of Otorhinolaryngology, Head and Neck Surgery, Prince of Wales Hospital, The Chinese University of Hong Kong, Shatin, Hong Kong SAR, China

**Keywords:** Cancer, Oncology

## Abstract

Multiportal video-assisted thoracic surgery (VATS) for major lung resection causes less immunochemokine production compared to thoracotomy. Whether uniportal VATS is similarly associated with lower early postoperative circulating levels of immunochemokines compared to multiportal VATS have not been studied. Selected patients who received uniportal or multiportal VATS major lung resection were recruited. Blood samples were collected preoperatively and on postoperative days 1 and 3 for enzyme linked immunosorbent assay of serum levels of Tissue Inhibitor of Metalloproteinase (TIMP)-1, Insulin Growth Factor Binding Protein (IGFBP)-3, and Matrix Metalloproteinase (MMP)-9. A linear mixed-effects models were used to analyze the effects of uniportal VATS on the postoperative circulating chemokine levels. From March 2014 to April 2017, 68 consecutive patients consented for the prospective study and received major lung resection by either uniportal VATS (N = 29) or multiportal VATS (N = 39) were identified. Uniportal VATS major lung resection was associated with lower post-operative levels of TIMP-1 and MMP-9 compared to multiportal VATS after controlling for the effects of the corresponding baseline level and the time of follow-up measurement. No difference was observed for the level of IGFBP-3. Less immunochemokine disturbances was observed after uniportal VATS major lung resection compared to multiportal VATS.

## Introduction

Attributed to the reduced access trauma, video-assisted thoracic surgery (VATS) has benefits over thoracotomy approach in terms of attenuated inflammatory cytokine response^1,2^, less disturbance of postoperative cellular immunity^3,4^, shorter hospital stay, less pulmonary^5^ and shoulder dysfunction following surgery^6^ and reduced postoperative pain^1^. Uniportal VATS has undergone rapid development and wide adoption over the past decade, constantly pushing the limits of minimally invasive thoracic surgery for lung cancer resection^7,8^. Studies have demonstrated uniportal VATS as a safe and feasible approach for major lung resection compared to the conventional multiportal approach, with some reporting additional benefits of reduced post-operative chest drain duration, length of stay, and morbidities^9^.

VATS major lung resection for non-small cell lung carcinoma (NSCLC) was known to be associated with differences in circulating levels of a number of chemokines, such as a higher circulating levels of insulin-like growth factor binding protein (IGFBP)-3, and lower levels of matrix metalloproteinase (MMP)-9 and tissue inhibitor of metalloproteinase (TIMP)-1, compared to the thoracotomy approach^10^. With the reduced length and number of wounds for uniportal VATS, further reduction in cytokine response following surgery may theoretically be detected; however, this has not been studied so far.

The objective of this prospective study was to examine the postoperative circulating levels of IGFBP-3, MMP-9 and TIMP-1 in patients undergoing major lung resection by uniportal VATS or multiportal VATS for malignant diseases.

## Materials and methods

### Operative strategy

Standardized preoperative investigations were performed including; 1) pathological diagnosis with fibreoptic bronchoscopy or, computer tomography-guided fine needle biopsy; 2) M-staging with positron emission tomography (PET); and 3) mediastinal staging with mediastinoscopy or endobronchial ultrasound whenever necessary. The results from histology were used for tumour (T), nodal (N), metastatic (M) staging. For uniportal VATS, an anterior 3–7 cm single utility thoracotomy without rib spreading was created in the 4th, 5th or 6th intercostal space^11^. For multiportal VATS, two additional 1–1.5 cm utility ports were created for camera and instrumentation. Major lung resection employs individual ligation technique, followed by mediastinal lymph node dissection according to the latest revised ESTS guidelines^12^. Both groups of patients received identical general anaesthesia with one lung ventilation. Intraoperative intercostal nerve block with 0.5% Levobupivacaine was given to both groups of patients at the end of the procedure. Pain control during early postoperative days was achieved by oral paracetamol, tramadol or dihydrocodeine. Additional non-steroidal anti-inflammatory drugs, gabapentin, or patient controlled analgesia may be used if pain control remained suboptimal.

### Data collection

The ethical approval is by the Joint Chinese University of Hong Kong-New Territories East Cluster Clinical Research Ethics Committee (Approval number: 2013.2.046). Informed consent for blood taking and analysis were complete in all participants. The medical records of consecutive patients with clinically resectable early-stage lung cancer or metastatic cancers who underwent VATS major lung resection were retrieved from patients’ electronic medical records. Data collected included preoperative patient demographic and comorbidities, disease status, operative procedures and postoperative outcomes. Patients were divided into two study groups: Uniportal VATS, and Multiportal (3-port) VATS. Patients were excluded from the study if they have: (1) long-term corticosteroid or immunosuppressant use; (2) diffuse dense pleural adhesions or complete pleural symphysis; (3) Utility thoracotomy longer than 7 cm; (4) conversion to open thoracotomy or required controlled rib fractures; or (5) declined consent. The primary outcome is the levels of IGFBP-3, MMP-9, and TIMP-1 on pre-operative, post-operative day 1 and post-operative day 3. The level of chemokines in patients with and without postoperative recurrence after curative resection for stage I adenocarcinoma were also compared.

Peripheral venous blood was collected in plain serum tubes 1 day before the operation as baseline, and at the same time on postoperative days (POD) 1 and 3. The sample was left to clot, and centrifuged at 3,000 rpm for 10 min at 4 °C. The serum was stored at -70 °C until assay. The concentrations of Insulin Growth Factor Binding Protein (IGFBP)-3, Matrix Metalloproteinase (MMP)-9, and Tissue Inhibitor of Metalloproteinase (TIMP)-1 were analysed by commercially available enzyme-linked immunosorbent assays kits (R&D Systems, Minneapolis, MN, USA), and were recorded by the same technologist blinded to the clinical data.

All methods were carried out in accordance with relevant guidelines and regulations.

### Statistical analysis

Descriptive statistics were used to compare the variable between the groups. Continuous variables following Normal distribution were reported as mean (± standard deviation). Continuous variables not following a Normal distribution were reported as median value (+ inter-quartile range). Categorical variables were reported as counts and percentages, and differences between two groups were assessed by Chi-square test, or Fisher’s exact test if a cell value was lower than 5. Descriptive statistics were analysed using the SPSS (Version 24.0, IBM, Armonk, NY, USA). A p-value < 0.05 represents statistically significant difference. Linear mixed-effects models were used to analyze the effects of multiportal VATS on the postoperative circulating levels of TIMP-1, IGFBP-3 and MMP-9. The models include the corresponding baseline level, the surgical approach (multiportal VATS or uniportal VATS), and the time of follow-up measurement (post-operative day 1 and day 3) as fixed effects, and the patient variation as a random effect.

The pre-operative and post-operative measurements of TIMP-1, IGFBP-3 and MMP-9 were not fully observed. The unrecorded entries are multiply imputed^13^ 100 times by chained equations using the R package “mice”^14^ upon assuming that the data were missing at random^15^. The mixed effect model is fitted by the R package “lme4”^16^.

## Results

A total of 68 consecutive patients fulfilling our inclusion criteria from March 2014 to April 2017 were included and divided into 2 groups: 29 patients in uniportal VATS group, and 39 patients in multiportal VATS group. The two cohorts have similar preoperative (Table 1), intraoperative characteristics and postoperative outcomes (Table 2). In particular, the incidence of diabetes, impaired renal function, and the operative time were similar between the groups. Both approaches are safe with no mortality, no need of conversion with additional ports, low incidence of minor complications, and similar chest drain duration and length of hospital stay. No patients required chemical pleurodesis during the first 3 days of postoperative stay, and no patients required intraoperative or postoperative blood transfusion. Primary adenocarcinoma of the lung is the commonest pathology in both groups, with 22 (75.9%) cases of uniportal VATS and 32 (82.1%) cases of multiportal VATS. Majority of the primary lung malignancies were stage I diseases (17/77.2% for uniportal VATS, and 27/71% for multiportal VATS) (Table 3).

**Table 1:** Preoperative parameters comparing Uniportal VATS versus Multiportal VATS.

Parameters	Uniportal VATS (N = 29)	Multiportal VATS (N = 39)	p-value
Male Sex	12 (41.4%)	20 (51.3%)	0.468
Age ≥ 65 years	9 (31%)	18 (46.2%)	0.208
Smoking	6 (20.7%)	14 (35.9%)	0.192
Old Tuberculosis	0	3 (7.7%)	0.255
Hypertension	8 (27.6%)	18 (46.2%)	0.119
Diabetes Mellitus	3 (10.3%)	12 (30.8%)	0.074
History of Malignancy	8 (27.6%)	13 (33.3%)	0.612
Previous Thoracic Surgery	1 (3.4%)	1 (2.6%)	1.000
Creatinine > 100 mmol/L	2 (6.9%)	9 (23.1%)	0.100
Forced expiratory volume in one second (% predicted)	98.7 ± 13.9	97.7 ± 22.8	0.837
Diffusing capacity for carbon monoxide (% predicted)	86.5 ± 22.2	78.4 ± 19.0	0.180

**Table 2:** Intraoperative and postoperative parameters comparing Uniportal VATS versus Multiportal VATS.

Parameters	Uniportal VATS (N = 29)	Multiportal VATS (N = 39)	p-value
Left-side Surgery	13 (44.8%)	15 (38.5%)	0.598
Upper lobe operation	12 (41.4%)	20 (51.3%)	0.418
Inferior pulmonary ligament released	28 (96.6%)	38 (97.4%)	1.000
Lymph node stations explored	3.9 ± 1.3	4.0 ± 1.4	0.803
Lung Sealants	5 (17.2%)	8 (20.5%)	1.000
Operative Time (minutes)	167.8 ± 52.8	187.2 ± 45.3	0.108
**Morbidity/Complications**	3 (10.3%)	10 (25.6%)	0.133
Persistent air leak > 5 days	1 (3.4%)	3 (7.7%)	0.631
Respiratory failure	0	2 (5.1%)	0.504
Acute retention of urine	1 (3.4%)	6 (15.4%)	0.225
Chest Drain Duration	3.3 ± 2.4	3.6 ± 2.0	0.586
Length of Stay	4.4 ± 2.6	4.6 ± 1.8	0.711

**Table 3:** Pathology of lung resections after Uniportal VATS versus Multiportal VATS.

Parameters	Uniportal VATS (N = 29)	Multiportal VATS (N = 39)	p-value
**Classify by stage**			
I	17	27	0.365
II	5	9	0.991
III	0	2	0.534
**Classify by histology**			
Primary lung adenocarcinoma	22 (75.9%)	32 (82.1%)	0.532
Others	7	7	
Squamous Cell Carcinoma	0	3	
Lymphoepithelial-like carcinoma	0	2	
Small cell carcinoma	1	0	
Metastatic	3	1	
Carcinoid	2	0	
Premalignant	1	0	

The results of cytokine analysis were shown in Table 4. Uniportal VATS major lung resection was associated with lower post-operative levels of TIMP-1 and MMP-9 compared to multiportal VATS. No differences were found for the level of IGFBP3.

**Table 4:** Post-operative serum immunochemokine level analysis after Uniportal VATS versus Multiportal VATS.

Immunochemokine (ng/mL)	Median (IQR)	Effect	Estimate	SE	FMI	p-value
**TIMP-1**		(Intercept)	44.85	13.62	0.203	**0.001**
Pre-operative	118 (103 – 144)	Baseline level	0.78	0.10	0.224	**0.000**
Post-operative day 1	131 (118 – 145)	Post-op day 3 versus post-op day 1	-1.56	5.36	0.211	0.772
Post-operative day 3	142 (118 – 167)	Uniportal versus multiportal VATS	-27.89	6.46	0.121	**0.000**
**IGFBP-3**		(Intercept)	552.35	193.56	0.083	**0.005**
Pre-operative	2119 (1511 – 2467)	Baseline level	0.91	0.08	0.086	**0.000**
Post-operative day 1	2323 (1814 – 3012)	Post-op day 3 versus post-op day 1	-211.35	85.04	0.195	**0.015**
Post-operative day 3	2233 (1626 – 2926)	Uniportal versus multiportal VATS	10.90	113.42	0.101	0.924
**MMP-9**		(Intercept)	1451.80	186.28	0.063	**0.000**
Pre-operative	481 (301 – 912)	Baseline level	0.13	0.12	0.121	0.265
Post-operative day 1	1258 (927 – 1762)	Post-op day 3 versus post-op day 1	-487.77	147.55	0.155	**0.001**
Post-operative day 3	912 (504 – 1398)	Uniportal versus multiportal VATS	-533.80	211.07	0.095	**0.013**

IQR: Interquartile range; Estimate: regression coefficient estimate; SE: standard error; FMI: fraction of missing information; IGFBP-3: Insulin Growth Factor Binding Protein-3; MMP-9: Matrix Metalloproteinase-9; TIMP-1: Tissue Inhibitor of Metalloproteinase-1.

Bold signifies significant *p*-value of <0.05.

## Discussion

The results of this study were mostly coherent with the results of our previous study on the level of MMP-9 and TIMP-1 comparing multiportal VATS and thoracotomy^10^, although we cannot demonstrate a statistically significant difference in the level of IGFBP-3. Nevertheless, our results similarly suggest that reduction in access trauma from uniportal VATS can lead to further attenuated immunochemokine responses.

For clinical stage I and II non-small cell lung cancer (NSCLC), VATS lobectomy might be better than thoracotomy in terms of long-term survival and recurrence outcomes although this remains controversial^5,17^. The difference in the levels of these cytokines remained one of the speculated possible mechanisms of the potential survival advantages following VATS major lung resection, apart from the attenuated cytokine-acute phase responses^1^ and better preserved immune function leading to improved tumour immunosurveillance^3,18^ with VATS. This survival benefits associated with minimally-invasive surgery was also seen in colorectal cancer^19^.

To explain, the extent of surgical access may partly explain the difference in the serum levels of insulin-like growth factor binding protein (IGFBP)-3^18^. Insulin growth factor (IGF)-1 is an important cell growth promoter and apoptosis preventer^20^. IGFBP-3 binds to and inhibits insulin growth factor (IGF)-1; can independently induce apoptosis in many colonic, prostatic, as well as in some NSCLC tumour cell lines^21,22^; and can also impair DNA synthesis in poorly-differentiated cancer cells^18^. Low circulating levels of IGFBP-3 have been linked to advanced prostate carcinoma^23^ and risk of developing colonic carcinoma^24^, oesophageal carcinoma^25^ and lung cancers^26^, and post-operative recurrence of colonic cancers^27^. Tumour cells may be shed into the circulation following lung cancer resection^28^, therefore the tumour-suppression properties of IGFBP-3 on these circulating tumour cells may be important.

Furthermore, surgical trauma has been associated with increased levels of matrix metalloproteinase (MMP)-9, which can deactivate IGFBP-3^18^. This mechanism was implicated in the tumorigenesis of MMP-7-producing IGF-IR-expressing primary tumours and to organ-specific metastasis^29^. MMP-9 possess proteolytic properties against type IV collagen within the basement membrane^30,31^, therefore might facilitate tumour invasion and metastasis in various tissues. TIMP-1, as its name implies, is a natural inhibitor of MMP. A lower serum level of TIMP-1 results in reduced inhibition on the action of MMP-9.

We observe that postoperative circulating levels of IGFBP-3 were similar following uniportal VATS major lung resection compared with the multiportal VATS approach. This may be explained by that the reduction in the size of surgical incisions in uniportal VATS was insignificant to produce a difference in levels of IGFBP-3. Furthermore, the blood levels of MMP-9, which can cleave and deactivate IGFBP-3, were reciprocally lower in the uniportal VATS group on postoperative day 1. Although the ESTS working group defined uniportal VATS when skin incision is ≤ 4 cm. The incision length for uniportal VATS could be longer due to many factors such as obesity or adhesions^32^.

One of the limitations of the study is the relatively low patient numbers, a result of limited voluntary participators, early discharge of patients leading to some missing data on postoperative day 3, and exclusion of significant number of patients which contribute towards confounding results (e.g. complete pleural symphysis, wound extension, open conversion and rib fractures). Future studies with larger sample size could look at immunochemokine levels in a more homogenous group, such as stage I primary lung cancer only. Another limitation is the non-randomized nature of the study. Although the demographics and pathology of the two groups of patients were comparable, and randomizing patients into uniportal and multiportal VATS groups may be difficult when patient’s choice preclude one or the other, our findings deserves to be validated in a randomized trial. Furthermore, quantifying local chemokine responses in the pleural cavity and lung tissue may be important in determining local tumour immunosurveillance. In addition, better preserved immune function following lung resection for cancer has yet to be shown to improve survival, particularly when other surgical and tumour related factors can have even more significant influence on outcome.

## Conclusion

Our study represents the first to investigate into the impact of uniportal VATS on circulating immunochemokine levels. Uniportal VATS major lung resection is associated with lower post-operative circulating levels of TIMP-1 and MMP-9, compared to the multiportal VATS approach, while the levels of circulating IGFBP-3 did not reach significant difference. The underlying mechanisms are likely multi-factorial, with access trauma playing an important role. The survival impact of these postoperative changes on tumor biology following lung resection for cancer warrants further investigation.
